# Development of Bioelectronic Devices Using Bionanohybrid Materials for Biocomputation System

**DOI:** 10.3390/mi10050347

**Published:** 2019-05-27

**Authors:** Jinho Yoon, Taek Lee, Jeong-Woo Choi

**Affiliations:** 1 Department of Chemical & Biomolecular Engineering, Sogang University, 35 Baekbeom-Ro, Mapo-Gu, Seoul 04107, Korea; iverson0607@naver.com; 2 Department of Chemical Engineering, Kwangwoon University, Wolgye-dong, Nowon-gu, Seoul 01899, Korea; tlee@kw.ac.kr

**Keywords:** bioelectronic devices, bionanohybrid material, biomemory, biologic gate, bioprocessor, protein, nucleic acid, nanoparticles

## Abstract

Bioelectronic devices have been researched widely because of their potential applications, such as information storage devices, biosensors, diagnosis systems, organism-mimicking processing system cell chips, and neural-mimicking systems. Introducing biomolecules including proteins, DNA, and RNA on silicon-based substrates has shown the powerful potential for granting various functional properties to chips, including specific functional electronic properties. Until now, to extend and improve their properties and performance, organic and inorganic materials such as graphene and gold nanoparticles have been combined with biomolecules. In particular, bionanohybrid materials that are composed of biomolecules and other materials have been researched because they can perform core roles of information storage and signal processing in bioelectronic devices using the unique properties derived from biomolecules. This review discusses bioelectronic devices related to computation systems such as biomemory, biologic gates, and bioprocessors based on bionanohybrid materials with a selective overview of recent research. This review contains a new direction for the development of bioelectronic devices to develop biocomputation systems using biomolecules in the future.

## 1. Introduction

Bioelectronics is defined as the combined field of biology and electronics that has recently been greatly developed to overcome the current limitation of silicon-based electronics and biology-based engineering [1]. By introducing biomolecules on the silicon-substrate, electrical functions have been demonstrated on the chip using the unique properties of biomolecules, such as specific target molecule detection and optoelectrical properties, that can be applied in bioelectronic devices such as biosensors, biophotodiodes, and biotransistors [2,3,4,5]. Various biomolecules including metalloprotein possess a metal ion at their core, and functional DNA with specific chemical group modifications such as amine and carboxyl groups have advantages for applications to develop bioelectronic devices because of their unique properties such as redox properties that are derived from the metal ion in the protein and the specific binding properties of DNA with its complementary DNA at the nanometer scale [6,7]. By fusing biomolecules with organic materials, electronic functions have been widely studied to develop bioelectronic devices with enhanced performance such as more sensitive target detection and increased signal [8,9,10,11]. Until now, many functional bioelectronic devices including protein-based bioelectronic chips that use the electron transfer mechanism of proteins and biophotodiode devices that use the photoelectric effect of rhodopsin have been reported [12,13,14]. However, current bioelectronic devices have certain critical limitations for practical application because the use of biomolecules inevitably accompanies limitations such as the low electrical/electrochemical signal-to-noise ratio derived from biomolecules, instability in harsh conditions, and narrow functionalization [15,16]. To overcome the limitations of biomolecules, innovative methods have been developed introducing nanoparticles to enhance the signal induced from biomolecules, combine biomolecules with carbon-based materials such as carbon nanotubes (CNT) or graphene for electrochemical signal increment and long-term stability using the biocompatibility of carbon-based materials, and the use of nanoscale-patterned chips as a platform for the extension of the functionality of bioelectronic devices such as by demonstrating nanoscale electronic functions and immobilizing different biomolecules independently at the nanometer scale to use these biomolecules simultaneously [17,18,19,20,21,22].

Recently, bionanohybrid materials composed of biomolecules and other nanomaterials have been developed widely for applications in bioelectronic devices. Bionanohybrid materials have received much attention for their wide application in developing delicate bioelectronic devices that accompany enhanced electronic functions or highly sensitive target detection for biosensors. As mentioned above, biomolecules have unique properties at the nanometer scale and nanomaterials such as nanoparticles, CNT, and biocompatible polymers that improve the properties of biomolecules can be hybridized precisely at the nanometer scale while retaining the properties of biomolecules and nanomaterials [23,24,25].

**Figure 1:**
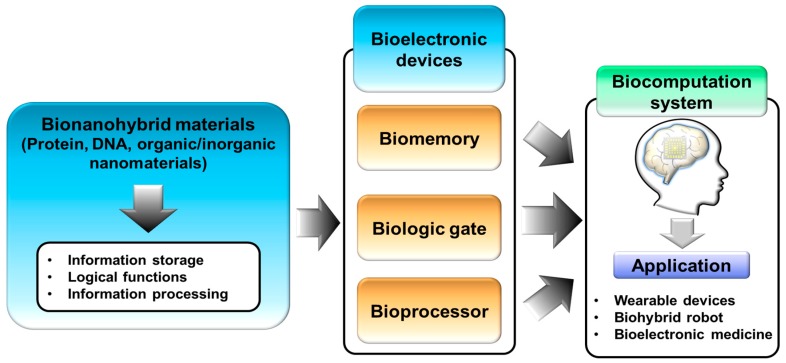
Bioelectronic devices based on bionanohybrid materials to develop biomemory, biologic gates, and bioprocessors for biocomputation systems.

Among the various bioelectronic devices, certain bioelectronic devices that are capable of performing information storage or signal processing similar to memory or logic gates in conventional electronic devices have shown a new perspective and direction for the development of biocomputation systems [26,27]. Biomemory devices based on metalloprotein or redox-controllable linker have been reported [28,29,30] that can demonstrate the memory function using biomolecules through controlling two apparently distinguished biomolecular states reversibly. In addition, using the above-mentioned bionanohybrid materials as the core component, bioprocessor devices have been reported that can process the input signal to process the out signal using bionanohybrid materials as the processing platform [31]. In addition, to develop sophisticated and improved functional bioelectronic devices, various advanced materials have been studied and introduced to fabricate modern devices such as new functionalized structural graphene and two-dimensional materials [32,33,34]. Through these efforts, various bionanohybrid materials that are capable of performing information storage, logical functions, and information processing have been developed for the development of bioelectronic devices including biomemory, biologic gates, and bioprocessors. Such bioelectronic devices can be used as core components to develop a biocomputation system that is capable of performing computation similar to conventional computers that are common in our surroundings as depicted in Figure 1.

In this review, bioelectronic devices based on bionanohybrid materials that are capable of performing information storage and signal processing for computation systems are discussed with a selective overview of recent research. Although there are many extensive reviews of bioelectronic devices, this review discusses in detail recent reports of various specific types of bioelectronic device for biocomputation systems. This review will suggest a new inspirable direction and aspects of bioelectronic devices to develop a biocomputation system [35,36].

## 2. Biomemory

Information storage is an important function for the operation of electronic devices. Until now, various information storage function devices have been developed in conventional silicon-based electronic devices through controlling two apparently distinguished states such as “1” and “0” states for the demonstration of conventional memory functions. From the bioelectronics perspective, some specific biomolecules have properties of existing in two distinguished states by external stimulation such as metal ion states that control metalloprotein, which can be utilized to develop biomolecular memory devices [37,38]. In addition, the hybridization of more than two types of biomolecule and bionanohybrid materials has been proposed to demonstrate multiple states control and increase the electrochemical signal derived from biomolecules for biomemory. In this chapter, we provide research related to biomemory devices including protein-based biomemory and resistive switching memory devices.

### 2.1. Multilevel Biomemory Devices

**Figure 2:**
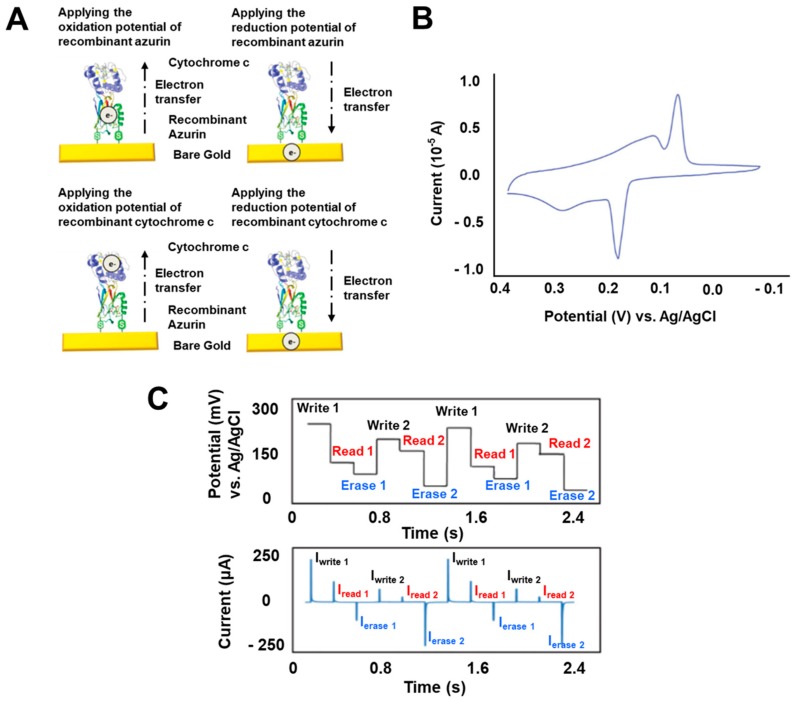
Multilevel biomemory device. (**A**) Schematic image demonstrating a multilevel biomemory device using metal ions states to control two different kinds of metalloprotein. (**B**) Cyclic voltammogram of a multilevel biomemory device composed of recombinant azurin and cytochrome c that shows two apparently distinguished reduction potential peaks and two oxidation potential peaks. (**C**) Memory performance of a multilevel biomemory device including writing, reading, and erasing steps by applying the potential values of reduction and oxidation potential peak values and the OCP values of metalloproteins. (Reproduced with permission from [43], published by John Wiley and Sons, 2010).

Metalloproteins have metal ions in their body that can be utilized for electrochemical investigation [39,40]. For example, the metal ion of a metalloprotein can be used to affect the redox reactions of specific materials, which can be measured using electrochemical techniques for developing biosensors [18,41]. In addition, this can be applied to develop biotransistors using redox properties [42]. This metal ion can exist in two different states like the Cu^+^ and Cu^2+^ states of azurin, a metalloprotein that possesses copper ion, which shows the potential of metalloprotein-based biomemory devices [28] by controlling metal ions with distinguished states. Various research groups have developed metalloprotein-based biomemory devices [29,37,38]. Among them, our group developed various biomemory devices using metalloproteins such as azurin and cytochrome c, which have never been reported before. Beyond just controlling the ion states of one type of metalloprotein for biomemory, we suggested multilevel biomemory devices using two kinds of metalloproteins to achieve the incremental memory density [43]. By controlling isoelectric points of metalloproteins via pH control, we immobilized two different metalloproteins, recombinant azurin modified with cysteine group and cytochrome c, directly on to the gold substrate by self-assembly through the electrostatic bond without any chemical linkers for the control of multiple redox states [44,45]. This simple immobilization process could reduce the immobilizing time of biomolecules, and remove the introduction of the other chemical materials for immobilization. Figure 2A shows the schematic image and demonstration of multilevel biomemory using the direct immobilization of two kinds of metalloproteins. We confirmed multilevel memory device fabrication by surface plasmon resonance (SPR) and scanning tunneling microscopy (STM) to verify the metalloprotein double layer formation through morphological changes. Then, an electrochemical investigation was performed using cyclic voltammetry (CV) and chronoamperometry (CA). By introducing two different metalloproteins, this device showed the multiple redox states that could be derived from copper ions of azurin and iron ions of cytochrome c as shown in Figure 2B. This showed oxidation potential peaks at 0.294 V and 0.184 V that were derived from cytochrome c and azurin, respectively, and the reduction of potential peaks at 0.131 V and 0.062 V from cytochrome c and azurin, respectively. These potential values for each metalloprotein were used as input potentials to control the metal ion states of the two metalloproteins. Then, we estimated the memory performance for this device using the obtained redox potential peak values of two metalloproteins for the “writing step” and “erasing step” and obtained the open circuit potential (OCP) values of metalloproteins for the “reading step” for multilevel biomemory demonstration. As shown in Figure 2C, this device showed apparently distinguished states when applying a potential to the device following expected schematic images with two different forms of “writing step”, “reading step”, and “erasing step”. From these results, we successfully developed new-concept multilevel biomemory devices using two different metalloproteins for multiple information storage biodevices.

### 2.2. Electrochemical Signal-Enhanced Biomemory Device

**Figure 3:**
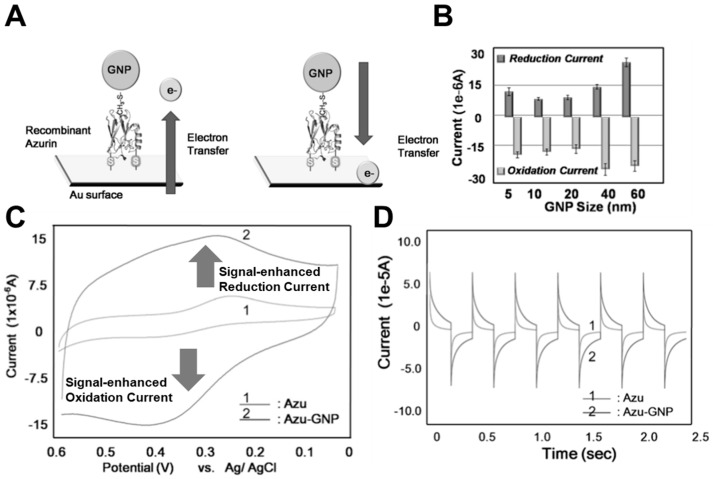
Electrochemical signal enhanced biomemory device. (**A**) Schematic image of the biomemory device composed of Azu and gold nanoparticles (GNP). (**B**) Redox potential peak values for optimizing the GNP diameter. (**C**) Cyclic voltammogram of Azu–GNP and Azu. (**D**) memory performance of Azu–GNP and Azu. (Reproduced with permission from [17], published by John Wiley and Sons, 2011).

As mentioned in the introduction, bioelectronic devices have certain limitations like the low electrical or electrochemical signal induced from biomolecules and low stability in harsh conditions [15,16]. To overcome these problems, various researchers have proposed the introduction of functional biocompatible nanomaterials for improved signal and stability [18,19,20]. Through these suggestions, biosensors and biofuel cells have been developed with advanced performance. In the case of biomemory devices, the extremely low electrochemical signal from biomolecules should be solved for application in practical applications. To achieve this, introducing metal nanoparticles can be a solution for signal enhancement. Gold nanoparticles (GNP) have been reported as an enhancer for the electron transfer reaction with metalloprotein [46]. Using the reported results, our group proposed a biomemory device using metalloprotein (azurin, Azu) and GNP to increase the electrochemical signal derived from metalloprotein (Figure 3A) [17]. To develop this electrochemical signal enhanced biomemory device, various GNP of nanometer size of diameter in the range 5–60 nm was introduced to find the optimized size for the GNP diameter. Based on the electrochemical signal increasing the redox potential peak values from CV results (Figure 3B), we found the optimized GNP size (5 nm) that showed smaller redox potential peak values compared to the results using the 60 nm GNP. However, in the case of the 60 nm GNP, the enhanced signal was not derived from Azu–GNP but directly induced from the immobilized GNP to the gold substrate without Azu. Therefore, the 5 nm GNP was chosen as the optimized size for biomemory fabrication. In addition, we assumed that the proposed increment of the electron transfer mechanism followed the equation below:

(1) $$GNP{\;}_{\overset{↔}{k_{-1}}}^{\;k_{1}}\;Protein{\;}_{\overset{↔}{k_{-2}}}^{\;k_{2}}\;Electrode$$

In Equation (1), *k*_1_ and *k*_−1_ are the electron transfer rate constants between the GNP and Azu and *k*_2_ and *k*_−2_ are the electron transfer rate constants between Azu and the gold substrate. By introducing the GNP, the electrochemical signal from Azu could be enhanced through the better electric coupling between azurin and GNP and between Azu and the gold substrate. Furthermore, the better coupling between Azu and GNP compared to that between Azu and the gold substrate induced a remarkably enhanced signal. After verifying the signal enhancement, the biomemory function of the proposed device was estimated. As shown in Figure 3C,D, the biomemory device composed of Azu and GNP (Azu–GNP) showed enhanced memory function compared to biomemory prepared with only Azu. The stored charge amounts were calculated by the following equation,

(2) Q = ∫i × dt

The current value (*i*) and time value (d*t*) were obtained by CA technique. To acquire the CA results, the redox potential peak values of Azu–GNP obtained by CV analysis were applied. From the calculation of the area underneath the CA graphs, the stored charge amounts of biomemory composed of Azu–GNP was about 4.503 µC, approximately four times higher than that of biomemory prepared with only Azu (about 1.1413 µC). This difference originated from the electric coupling between Azu and the GNP. Through this research, electrochemical signal-enhanced biomemory was developed for the first time, and this approach may demonstrate the possibility of developing accurate nanoscale biomemory devices that can overcome the problems associated with low electrochemical signals.

### 2.3. Resistive Biomemory Device

**Figure 4:**
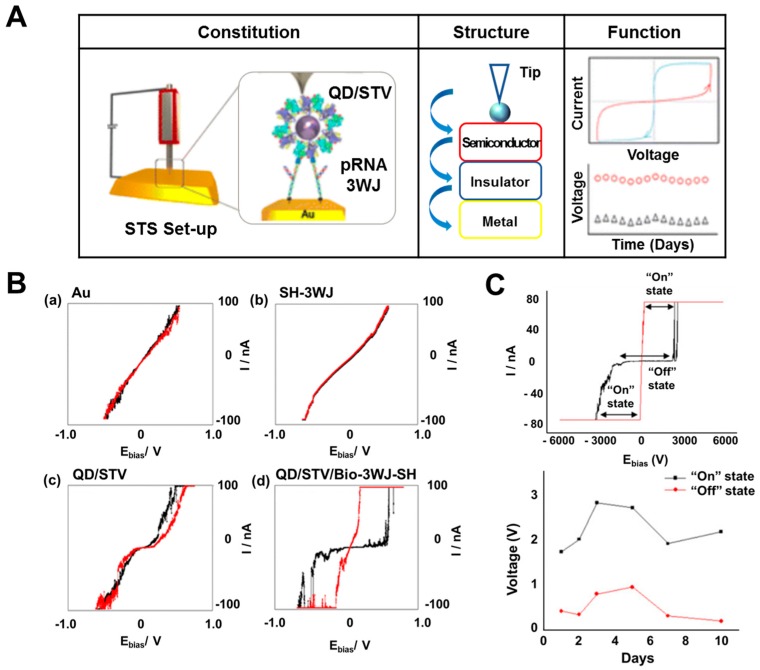
Resistive biomemory device. (**A**) Schematic image of a resistive biomemory device composed of pRNA-3WJ and quantum dot (QD) on a gold substrate, (**B**) I–V curves of bare Au, pRNA-3WJ, QD and pRNA-3WJ, and QD. (**C**) Resistive switching function and stability test for a resistive biomemory device composed of MoS_2_ and DNA on a gold substrate with apparently distinguished resistance states and long-term stability for 10 days. (Reproduced with permission from [50], published by the American Chemical Society, 2015, and reproduced with permission from [52], published by Elsevier, 2019).

In conventional silicon-based electronic fields, huge attention has been paid to the development of resistive memory devices for resistive switching function demonstration. Resistive memory devices have been researched widely for commercialization due to their advantages such as fast processing and response and low energy requirement. In the case of the existence of metal–insulator–metal layers or semiconductor–insulator–metal layers on the substrate, there are specific unique hysteresis properties at some voltage range with two apparently different resistance values (extremely high resistance value and extremely low resistance value) following theories such as ohmic conduction, thermionic emission, Schottky emission, or tunneling current [47]. Various research groups have developed organic material-based resistive memory devices [48,49]. Biomolecules are suitable for demonstrating resistive switching functionality at the nanometer scale because they possess unique properties at such scale. Guo’s group developed a resistive biomemory device using the RNA structure and quantum dot (QD) by collaboration with our group [50]. In previous research, they developed a packaging RNA (pRNA) three-way junction structure (pRNA-3WJ) that showed thermodynamically stability [51]. This pRNA-3WJ could overcome the critical limitations of conventional RNA such as extremely low stability even at room temperature. In resistive biomemory research, they introduced the developed pRNA-3WJ as a stable insulator to demonstrate the resistive switching function. Figure 4A shows the schematic images and resistive function in this device. Using the biological binding properties between streptavidin and biotin, they developed a nanoscale bionanohybrid material composed of pRNA-3WJ and QD. The conjugation of pRNA-3WJ and QD for bionanohybrid materials was verified by electrophoresis through the existence of the upper located band due to the increased total weight and size by QD introduction compared to the band in only pRNA-3WJ without QD. After immobilizing this bionanohybrid material on the gold substrate, pRNA-3WJ performed a role as an insulating layer and QD as the semiconducting layer on the conducting gold layer. Using a scanning tunneling spectroscopy (STS) technique, they estimated the resistive switching function of this device at the nanometer scale using the platinum tip as the probe located on this bionanohybrid material. Figure 4B displays the I–V curve of a bionanohybrid material on a gold substrate. Compared to the gold substrate alone, only pRNA-3WJ, and only QD on a gold substrate, a bionanohybrid material composed of pRNA-3WJ and QD on a gold substrate showed apparently distinguished resistance values with extremely high and low resistance at the voltage range of +3 to −3 V. This bistable behavior could be defined as “On state” and “Off state” for resistive memory applications.

Our group also developed a resistive biomemory device based on two-dimensional material. A bionanohybrid material composed of molybdenum disulfide nanoparticles (MoS_2_) and a DNA layer on a gold substrate was developed to demonstrate resistive switching functionality at the nanometer scale [52]. To develop this resistive biomemory at the nanometer scale, we immobilized DNA and synthesized MoS_2_ sequentially on a complementary DNA modified gold substrate. Then, a semiconductor (MoS_2_)–insulator (DNA)–metal layer (gold substrate) was formed that could demonstrate resistive switching functionality through specific unique hysteresis properties at a certain voltage range with two apparently different resistance values. MoS_2_ is a metal dichalcogenide material that has been widely used to develop bioelectronic devices because of its unique properties including biocompatibility, excellent semiconductivity, and its optical properties [53,54]. To demonstrate resistive switching functionality at the nanometer scale, MoS_2_ nanoparticles with surface modification (carboxyl group) was synthesized for the first time to conjugate efficiently with amine-tagged DNA via EDC/NHS bonding. The synthesis of surface-modified MoS_2_ nanoparticles and the fabrication of bionanohybrid materials were verified by transmission electron microscopy (TEM) for MoS_2_ synthesized nanoparticles, energy-dispersive X-ray spectroscopy (EDS) for elemental analysis, electrophoresis for the conjugation of MoS_2_ and DNA, and STM techniques to immobilize this bionanohybrid material on the gold substrate. Using STS analysis, the proposed resistive biomemory device based on MoS_2_ and DNA showed the resistive switching function with bistable states at a wide voltage range (4 to −4 V) and long-term stability as shown in Figure 4C. Figure 4C shows that the resistance value dramatically decreased when the voltage reached 2.4 V; on the other hand, the resistance value abruptly increased when the voltage reached 0.01 V. In addition, by introducing DNA as the insulating layer, which is more stable than RNA, it showed resistive switching function for about 10 days. As with these studies, bionanohybrid material-based resistive biomemory devices have been researched to demonstrate resistive switching functionality at the nanometer scale, which suggests a future direction for the development of the next generation of memory devices using biomolecules.

## 3. Biologic Gate

Among the various components of computing systems, logic gates are a core component in the computing process. These electrical circuits implement Boolean functions that can perform logical operations by converting more than two inputs to one binary output. Until now, many logic gates have been developed including AND (the gate that performing the logical conjugation), OR (the gate that performing the logical disjunction), NOT (the gate that performing the logical negation), XOR (the gate that giving the true outputted signal when the number of true inputs is odd), and NAND (the gate that giving the false outputted signal only when all inputs are true) gates [55,56]. In bioelectronics, some biomolecules can interact with specific chemical materials or biomolecules. For example, myoglobin can react with hydrogen peroxide [18] and glucose oxidase can react with glucose [57]. These properties can be utilized to demonstrate a logic gate using biomolecular interactions by controlling the input materials. In addition, conformational changes of biomolecules can be utilized to develop logic gates such as the conformational change of G-quadruplex DNA (G-rich DNA) as a bending shape and straightening shape that is dependent on the pH value [58]. Furthermore, these logic functions based on biomolecules can provide opportunities to mimic the analog human decision-making process [59] through controlling the combination of biomolecules and organic and inorganic materials. In this chapter, we will introduce research into biologic gates using biomolecules such as proteins and DNA and bioelectronic devices that mimic the analog human decision-making process.

### 3.1. DNA-Based Biologic Gate

**Figure 5:**
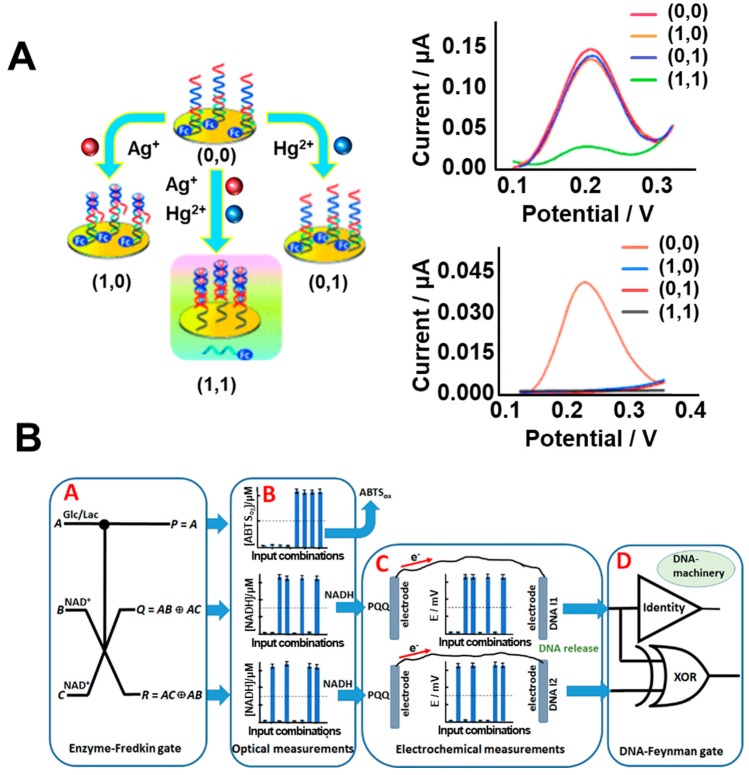
Biologic gates. (**A**) Schematic image of a DNA-based biologic gate based on metal ions inserted inside mismatched DNA pairs and differential pulse voltammetry (DPV) results of this device by controlling output signals through Ag^+^ and Hg^2+^ ions inserted inside mismatched DNA pairs. (**B**) Schematic image of a protein/DNA-based biologic gate through the signal transduction of a protein-based biologic gate to a DNA-based biologic gate for the final outputted fluorescence signal (Reproduced with permission from [64], published by John Wiley and Sons, 2013 and reproduced with permission from [65], published by John Wiley and Sons, 2016).

DNA is the smallest level at which the composition of living organisms is developed. There have been reports related to DNA research such as DNA sequencing, immunoassay, and DNA structure formation for wider applications [60,61,62]. From the bioelectronics perspective, the unique properties of DNA have received attention for their granting of functionality to bioelectronic devices. DNA can specifically bind with complementary DNA and the structure of DNA can be controlled by external responses [58]. Until now, various DNA-based logic gates have been reported based on colorimetric or fluorescence investigations. However, electrochemical techniques are better suited to bioelectronic device fabrication due to their fast response, minimal required reagents, and simplified outputs compared to colorimetric-based bioelectronic devices. In addition, a report found that mismatched sequences of double strand DNA such as cytosine–cytosine (C–C) and thymine–thymine (T–T) mismatched pairs could possess metal ions in such locations [63]. From this perspective, Qiu’s group developed a biologic gate using this unique property of DNA [64]. Figure 5A showed the schematic process and results of AND logic gates based on DNA mismatching. Silver ions (Ag^+^) and mercury ions (Hg^2+^) could enter the mismatched C–C and T–T pairs, respectively. Using these properties, they designed T- and C-rich DNA sequences with ferrocenecarboxylic acid (Fc) as the redox generator. In this device, metal ions were used as input molecules and the electrochemical signal from Fc was the output signal from the logic gate. By controlling the DNA sequences, they developed AND, NAND, and NOR logic gates through controlling the output signal using the unique electrochemical signals derived from the inserted Ag^+^ and Hg^2+^ ions located inside the mismatched pairs in the DNA. From the results, in the case of the insertion of only both Ag^+^ and Hg^2+^ ions, the electrochemical signal was detected by the differential pulse voltammetry (DPV), which was defined as “1” due to the co-existence of Ag^+^ and Hg^2+^ ions as shown in Figure 5A. Furthermore, this logic gate based on DNA mismatching can be operated reversibly compared to DNA cleavage-based logic gates. This result showed the possibility of applying bionanohybrid materials based on specifically designed DNA sequences and metal ions for both bioelectronic devices and for the development of a multiplexed biosensing platform.

### 3.2. Protein/DNA-Based Biologic Gate

Their distinct properties mean that proteins are widely utilized in bioelectronics. As mentioned in the introduction, proteins have certain unique advantages such as distinctive redox properties that can target specific molecules and reactions. Through these properties, protein-based biologic gates, particularly enzyme-based biologic gates, have been broadly developed [66,67]. Aida’s group developed a biologic gate by protein folding [68] and Schöning’s group proposed a biologic gate using a membrane composed of multiple enzymes [69]. Recently, Katz’s group developed reversible biologic gates based on both enzymes and DNA [65]. They developed and combined an enzyme-based biologic gate with a reversible DNA-based biologic gate through a biomolecular electrode to create complex reversible logical computing systems. This proposed system was composed of an enzyme-based Fredkin gate that was capable of converting three input signals to three output signals and a DNA-based Feynman gate that was capable of converting two input signals to two output signals [70,71]. To demonstrate this complex biologic gate, they introduced the optical, electrochemical, and fluorescent measurement techniques. Figure 5B shows a schematic diagram of this complex biologic system that is composed of a protein-based biologic gate and a DNA-based biologic gate as demonstrated by the enzyme reaction and connected DNA reaction. Glucose (Glc), lactic acid (Lac), and β-nicotinamide adenine dinucleotide hydrate (NAD^+^) were used as three input signals for the enzyme-based Fredkin biologic gate and glucose dehydrogenase (GDH), lactate dehydrogenase (LDH), glucose oxidase (GOx), and horseradish peroxidase (HRP) were utilized for enzyme-based biologic operation. After reacting in the first enzyme-based biologic gate, the generated signal that produced NADH through enzymatic reactions was measured by an optical technique and transferred to the electrochemical system for electrochemical enzymatic reaction. Then, in the final stage at the connected DNA-based biologic gate, the transferred signal was converted to fluorescent final outputs. Pyrroloquinoline quinone (PQQ)-modified electrode and iron ion (Fe^3+^) crosslinked alginate-modified electrode with entrapped DNA were used for the electrochemical system and final DNA-based biologic system. By the electrochemical reaction, the Fe^3+^ ion of the crosslinked alginate-modified electrode with entrapped DNA was oxidized to Fe^2+^ and the entrapped DNA was released from the alginate-modified electrode to a DNA-based biologic gate for the final fluorescence output signal. Although many components and complex biological reactions were utilized for this biologic gate, they developed a complex biologic system that was composed of two different kinds of biomolecule-based biologic gates that were more complex than the reported biologic gate to accurately mimic a conventional silicon-based electronic logic system.

### 3.3. Analog Decision Mimicking Bioelectronic Device

**Figure 6:**
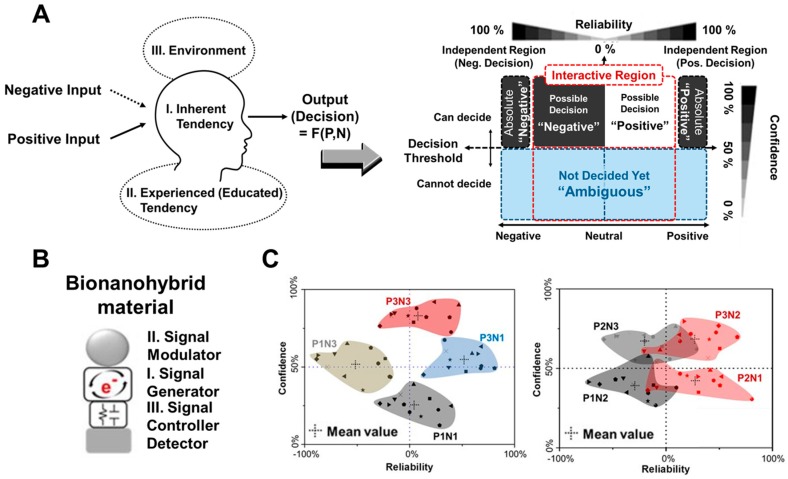
Analog decision mimicking bioelectronic device. (**A**) Schematic image and theory of this bioelectronic device through the analogously processed output signals by two different external factors inputted (negative input and positive input) by electrochemical investigation. (**B**) Bionanohybrid material used for this device composed of metalloprotein used as signal generator, organic chemical linkers as signal controller, and inorganic materials used for signal modulation. (**C**) The plotted results of analog decision-making based on the analysis of electrochemical signal by defined external factors showed the decision variation of 12 persons based on the defined threshold values. (Reproduced with permission from [59], the figures follow the terms of use under a Creative Commons Attribution 4.0 International License.).

In conventional silicon-based electronics, only digitalized processing, logic, and arithmetic operations have been developed and utilized in all devices [72,73]. These operations have certain advantages for the development of electronic devices in which the binary coded digital signals “1” and “0” can be distinguished, defined, and operated easily by converting input signals into an integrated single output signal. However, digital signal-based conventional electronic devices have limitations for the demonstration of human logic systems or other analog decision-making processes because these systems are not decided or operated by one simple and single digital input and output, but are instead affected by a myriad of complex factors such as personality, experience, and intelligence. The critical difference between conventional electronic devices and real human logic systems can hinder the development of biocomputation systems. Therefore, in bioelectronic fields, there have been studies to develop bioelectronic devices that are capable of mimicking analog decisions or analog logic systems [74] by considering various factors for analog calculation. Liu’s group developed four analog computing systems and extended the range of computing to real numbers based on DNA by connecting DNA-based biologic gates using unique properties of DNA such as DNA strand displacement. This result showed the potential of DNA-based real number calculation such as a calculator and by extension a combination of various DNA-based biologic gates; this could demonstrate more complex number calculation. In addition, bioelectronic noses and tongues based on biomolecular receptors have been researched recently to mimic the processes of real living organisms [75,76]. To demonstrate the analog decision-making process on a bioelectronic chip, our group developed an electrochemical bioelectronic device based on a bionanohybrid material composed of metalloprotein and organic/inorganic nanomaterials or metal ions [59]. Figure 6A shows a conceptual image of their research for mimicking analog decision-making through the analogously processed output signals by inputting two different external factors (negative input and positive input) via electrochemical investigation. We defined specific regions of the acquired signal as the degree of confidence and reliability of a human following defined threshold values. Myoglobin (Mb) that is a metalloprotein used as signal generator and defined as an inherent human tendency, organic chemical linkers that are used as signal controllers and defined as experience-induced human tendencies, and inorganic materials that are used for signal modulation and defined as environment-dependent signal modulators were combined to demonstrate analog decision-making by signal control and modulation (Figure 6B). As shown in Figure 6C, the plotted results of analog decision-making based on the analysis of electrochemical signals by defined external factors showed the decision variation of 12 people based on defined threshold values. This research shows the conceptual potential for the development of analog-based bioelectronic devices which has never been reported to apply for biocomputation systems. Of course, many subjective definitions exist that can demonstrate the analog decision-making process on a bioelectronic chip. This research shows one potential development route for a human mimicking analog computation system.

## 4. Bioprocessor

Until now, numerous molecular electronic devices have been developed to miniaturize electronic devices at the molecular scale for overcoming the physical or technological limitations of conventional silicon-based electronic devices, such as difficulty to achieve compact integration at nanometer or molecular scale [77]. Biomolecules have unique properties even at the nanometer scale that are suitable to complement the molecular electronic devices with delicate functional processing properties. Accordingly, some researchers developed bioprocessors that could control the biological output signals, such as the expressed gene level, through the biological reaction process by specific inputted biomolecules [78,79]. To mimic the conventional silicon-based electronic processors, especially, the functional bionanohybrid material composed of biomolecules and various nanomaterials can used for processing the input signal converted to the processed output signal such as electrochemical signal. In addition, specifically designed microchips can be a powerful tool to control the biologically processed output signals through the control of biological reactions. In this chapter, we will provide the recently developed bioprocessor devices that could mimic the processing in electronic devices.

### 4.1. DNA-Based Bioprocessor

**Figure 7:**
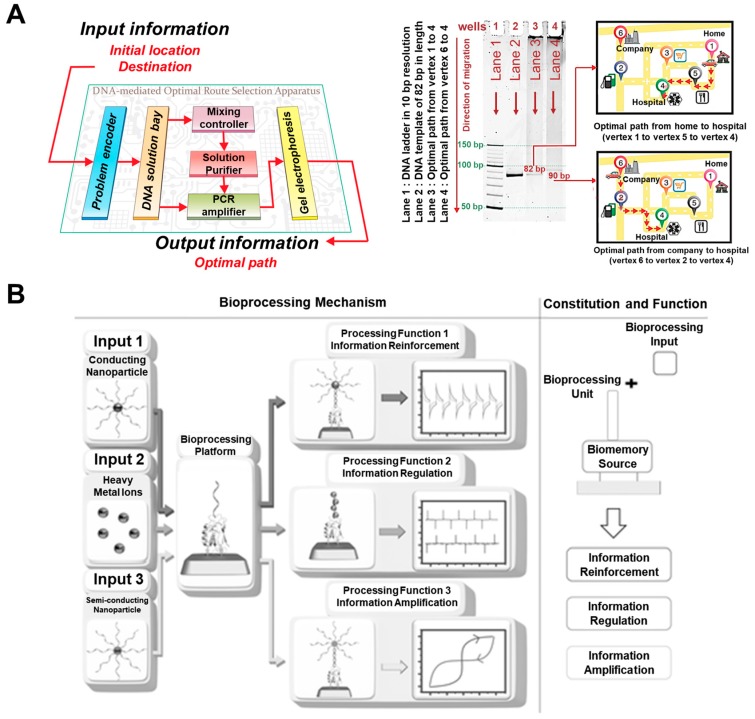
Bioprocessors. (**A**) Schematic image and electrophoresis results of DNA-based bioprocessor composed of six stages, including the first stage as problem encoder, the second stage as DNA solution bay for converted DNA preparation, the third as mixing controller for mixing and ligase of appropriate DNA sequences to make the template of DNA duplexes, the fourth as solution purifier for isolation of optimal DNA template from impurities such as the incompletely hybridized oligonucleotides or enzymes, the fifth as PCR amplifier for amplification of optimal DNA template which is the optimal route, and the sixth as gel electrophoresis to acquire the final electrophoresis data for solving optimal-route-planning problems, (**B**) Schematic image of bioprocessor based on bionanohybrid materials to demonstrate the specific processing functions including the electrochemical signal reinforcement, regulation, and amplification. (Reproduced with permission from [82], published by American Chemical Society, 2015, and reproduced with permission from [31], published by John Wiley and Sons, 2013).

As mentioned in the above chapters, DNA is a suitable biomaterial for bioelectronic applications. Especially, the massive parallelism of DNA hybridization exhibits tremendous potential, which can be utilized to develop feasible electronic devices capable of performing processing or computing operation to fulfill the demands of monolithic parallel computing system with specific computational algorithm [80]. From this point of view, DNA-based bioprocessors or applications have been reported [81]. Lee’s group proposed a novel programmable DNA-mediated processor to solve the optimal route planning problems [82]. To achieve this functional DNA-mediated bioprocessor, they fabricated the programmable optimal route planning apparatus comprising six stages, as shown in Figure 7A. Also, the routes shown in Figure 7A were defined following the specific DNA sequences (20mer single strand DNA) to find the optimal route based on DNA processing through the conventional PCR reaction using the inputted DNA sequences, which determine distance between specific locations as shown in the map of the right side of Figure 7A. To operate this PCR system, they defined the first stage as problem encoder for conversion of vertices and weighted edges of the designed route to DNA sequences, and all distances between each of the six locations were defined as specific DNA sequences (20mer single strand DNA) to apply for the program encoder. They defined the second stage as DNA solution bay for converted DNA preparation, the third as mixing controller for mixing and ligase of appropriate DNA sequences to make the template of DNA duplexes that represents the possible routes, the fourth as solution purifier for isolation of optimal DNA template from impurities such as the incompletely hybridized oligonucleotides or enzymes, and the fifth as PCR amplifier for amplification of optimal DNA template which is the optimal route, final as gel electrophoresis to acquire the final electrophoresis data for optimal DNA template as the find of the defined optimal route. Using these stages for optimal-route finding, they performed the DNA reactions at these mentioned stages by binding and amplification of the combined six defined DNA sequences for six locations. They obtained the results of electrophoresis to find the optimal route from home or company to the hospital as shown in Figure 7A using subjectively defined factors. Although there existed too many subjective definitions for operation, these results showed the possibility of DNA-based bioprocessing for solving the practical problems; this could be demonstrated with much fewer components and materials compared to the conventional silicon-based electronic devices. Until now, DNA-based bioprocessors remain at the early stage. However, due to the huge researches for DNA-based bioprocessors, the more sophisticate and functional processable bioprocessors will be developed.

### 4.2. Bioprocessor Based on Bionanohybrid Material

Advancing from only DNA-based bioprocessors, bionanohybrid material based on DNA can be used as the platform to develop the bioprocessor with more intuitive bioprocessing operation, without too much subjective definition seen in DNA-based bioprocessors, such as definition of the specific DNA sequences as the specific distance between home or company to hospital for solving the optimal-route finding. Our group developed the bioprocessing device based on bionanohybrid materials composed of protein, DNA, and inorganic nanomaterials to demonstrate the various bioprocessing functions using electrochemical/electrical investigation [31]. To develop this bioprocessor, the recombinant protein (azurin, Azu) and single-strand DNA were conjugated through the organic linker as the electrochemical signal generating bioprocessing unit (Azu/DNA hybrid) by the redox properties derived from recombinant azurin. Then, the complementary DNA (cDNA) and gold nanoparticle (GNP) hybrid (cDNA/GNP), heavy metal ions, and cDNA and quantum dot (QD) hybrid (cDNA/QD) were introduced to the bioprocessing unit as the input materials for electrochemical signal reinforcement, regulation, and amplification. Figure 7B showed the schematic image of this bioprocessor, which processed the three different outputs by introduced each input material. In the case of cDNA/GNP introduction, the electrochemical signal from bioprocessing unit was reinforced by the existence of conducting GNP. Moreover, in the case of introduction of heavy metal ions, the electrochemical signal was regulated by existed heavy metal ions such as Cu, Zn, Ni, Co, Fe, and Mn through the movement of redox peak values compared with the peak values of only Azu/DNA hybrid without metal ions. In addition, in the case of introduction of the cDNA/QD as the semiconducting nanoparticle to the bioprocessing unit, the processed electrical signal showed the electrical bistable properties as the resistive memory function by STS investigation compared to the result of only the bioprocessing unit without cDNA/QD. This developed bioprocessor device can process three different functions in the single bionanohybrid material using electrochemical and electrical signals intuitively compared to the bioprocessors demonstrated based on subjective definitions. It showed the possibility of the development of the biocomputation system in a single-biomolecular hybrid at nanometer scale.

## 5. Future Perspective

Since the 1960s, silicon-based electronic devices have been developed widely to demonstrate more complex functions with faster and more efficient processing on nanoscale-size chips. However, until now, the demonstration of a computation system on the single-molecular level has been impossible in the electronics field. To develop the single-molecular computation system, bioelectronic devices present new possibilities in the development of single biomolecular computation systems based on bionanohybrid materials. Bionanohybrid materials composed of biomolecules such as protein or DNA, and organic/inorganic nanomaterials can perform sophisticated functions at the single-biomolecular level to apply for bioelectronic devices. In this review, authors discussed the various research areas related to the bioelectronic devices including biomemory, biologic gates, and bioprocessors, which are the core components of the computation system. First, we discussed biomemory device based on the metalloprotein heterolayer, metalloprotein-nanoparticle hybrids, and nucleic acids-semiconducting nanoparticle hybrids. To achieve the memory function, developed bionanohybrid materials should demonstrate the two distinctive bistable states, which can be defined as ‘1’ and ‘0’ states for memory. As shown in results, those bionanohybrid materials showed apparently distinguished bistable states by electrochemical or electrical investigation. Next, we examined the studies about biologic gates based on the DNA–metal ion hybrids, protein–DNA connected reaction, and protein–organic/inorganic nanomaterial hybrids. Using these bionanohybrid material, various logic functions including the AND, NAND, Fredkin, or Feynman logic gates were demonstrated. Furthermore, the human analog decision-mimicking device was developed. In addition, we discussed about bioprocessors capable of processing of the inputted signals to the output signals such as finding of optimal routes and processing of different electrochemical signals through the DNA reactions and metalloprotein, DNA and inorganic nanomaterial hybrids. In addition to the results discussed in this review, many research groups have studied to develop the delicate functional bionanohybrid materials to apply for biomemory, biologic gates, and bioprocessors. The bioelectronic devices comprised with bionanohybrid materials would be a milestone for biomolecular-computation systems in the near future. Moreover, this will provide a useful way of bioelectronic devices to apply in development of wearable devices [83,84], biohybrid robots [85,86,87], and bioelectronic medicine [88,89].
